# HLA-E: A Novel Player for Histocompatibility

**DOI:** 10.1155/2014/352160

**Published:** 2014-10-20

**Authors:** Thomas Kraemer, Rainer Blasczyk, Christina Bade-Doeding

**Affiliations:** Institute for Transfusion Medicine, Hannover Medical School, Medical Park, Feodor-Lynen-Straße 5, 30625 Hannover, Germany

## Abstract

The classical class I human leukocyte antigens (HLA-A, -B, and -C) present allele-specific self- or pathogenic peptides originated by intracellular processing to CD8^+^ immune effector cells. Even a single mismatch in the heavy chain (hc) of an HLA class I molecule can impact on the peptide binding profile. Since HLA class I molecules are highly polymorphic and most of their polymorphisms affect the peptide binding region (PBR), it becomes obvious that systematic HLA matching is crucial in determining the outcome of transplantation. The opposite holds true for the nonclassical HLA class I molecule HLA-E. HLA-E polymorphism is restricted to two functional versions and is thought to present a limited set of highly conserved peptides derived from class I leader sequences. However, HLA-E appears to be a ligand for the innate and adaptive immune system, where the immunological response to peptide-HLA-E complexes is dictated through the sequence of the bound peptide. Structural investigations clearly demonstrate how subtle amino acid differences impact the strength and response of the cognate CD94/NKG2 or T cell receptor.

## 1. Introduction

The few polymorphic HLA-E alleles are restricted to two functional variants HLA-E^*^01 : 01 and HLA-E^*^01 : 03. Officially, there are 13 HLA-E alleles recognized by the International Immunogenetics Database to date; however, only HLA-E^*^01 : 01 and ^*^01 : 03 contribute to HLA-E function [1]. These two alleles are distributed almost equally among diverse populations. The maintenance of these two alleles is most likely based on a balancing selection, meaning that there is a heterozygote advantage for individuals that are heterozygous at the HLA-E gene locus [2]. This is in contrast to the classical HLA molecules that possess high frequencies of polymorphisms with crucial functional differences, maintained by overdominant selection [3].

The polymorphisms can be maintained by selection favouring the heterozygote genotype. In classical HLA class I molecules these polymorphisms impact on antigen presentation, such as alteration of peptide binding motifs [5–7] that result in modification of the whole peptide/HLA landscape [8]. HLA-E^*^01 : 01 and HLA-E^*^01 : 03 differ exclusively in one amino acid (AA) substitution at position 107, located on a loop between *β*-strands in the *α*2 domain of the heavy chain (hc), where an arginine for HLA-E^*^01 : 01 is substituted by a glycine for HLA-E^*^01 : 03. A substitution at this position is unlikely influencing peptide presentation, because it is not located in the eight stranded *β*-pleated sheet, or in the peptide binding groove's *α*-helix segments [9]; however, structural impact on the proximate AAs located within the peptide binding cleft cannot be excluded [10]. Nevertheless, the conservation of the two functional HLA-E alleles is equally among populations, concluding a likely functional difference [11, 12]. Evidence for this hypothesis is the difference in surface expression of HLA-E^*^01 : 01 and HLA-E^*^01 : 03. The surface expression of HLA-E^*^01 : 01 is reported to be significantly lower compared to HLA-E^*^01 : 03 on human lymphoblastic B cell lines (B-LCL) [10]. Consistent with the differences in surface expression thermal stability studies revealed that HLA-E^*^01 : 03 molecules have a higher thermal stability compared to HLA-E^*^01 : 01 molecules that were refolded in the presence of the same HLA class I signal peptide [10].

The binding of a peptide to HLA-E provides a stable trimeric complex that is presented on the cell surface. The main source of HLA-E peptide ligands is signal peptides derived from classical HLA molecules [13, 14]. The relative overall surface expression of both alleles is influenced by the combination of the present HLA-A, -B, and -C allotypes in individuals, since the cell surface expression and stability of HLA-E are based on the available HLA class I derived signal peptides [15].

As the HLA-E peptidome was first thought to be restricted to peptides derived from this source,* in vitro* studies with random peptide libraries have shown that HLA-E is capable of binding a range of different peptides and is not only restricted to peptides derived from classical HLA molecules [16], although a preference for hydrophobic residues at most positions of the peptide is evidenced. The range of HLA-E peptide selection includes the identified peptide ligand QMRPVSRVL derived from the HSP60 protein that upregulates HLA-E surface expression due to cellular stress response [17]. Additionally, peptide ligands with distinct differences in their AA sequence have been shown to bind to HLA-E. A peptide derived from the ATP-binding cassette transporter, multidrug resistance-associated protein 7 (MRP7) ALALVRMLI was identified to bind HLA-E during heat shock [18]; the peptide AISPRTLNA derived from the HIV Gag protein has been shown to upregulate HLA-E surface expression on HIV infected lymphocytes [19]. A peptide SQQPYLQLQ derived from gliadin, that is, the known antigen for priming the celiac disease pathogenesis, stabilizes HLA-E levels in celiac patients [20]. The HCV core35–44 peptide YLLPRRGPRL stabilized the HLA-E complex and conferred protection against NK cell mediated lysis through specific interaction with the CD94/NKG2A receptor [21]. Recent studies investigated the HLA-E derived peptide repertoire and confirmed striking differences in their anchor position and features [22]. In any case, the broadened peptide ligand varieties (Table 1) and functional potential of HLA-E gained more attention.

## 2. Regulatory Interactions of pHLA-E Complexes

HLA-E is a mediator of NK cell activation and inhibition [23, 24]. Peptide-HLA-E (pHLA-E) complexes are ligands for either the inhibitory CD94/NKG2A or the stimulatory CD94/NKG2C NK cell receptor. The importance of the peptide sequence for the interaction with these receptors needs to be emphasized in this respect. Structural studies on the interaction between the CD94/NKG2 heterodimer and HLA-E are based on a pHLA-E complex bound to a peptide VMAPRTLFL derived from the HLA-G leader sequence (PDB: 3CDG). The affinity of HLA-E^VMAPRTLFL^ to the CD94/NKG2A receptor is approximately 6 times higher than to the CD94/NKG2C receptor, as a result of differential interactions between distinct residues of either CD94 or NKG2 with particular residues on the HLA-E *α*1 helix and residues within the peptide [4, 25]. The distinct difference in affinity could also be verified with another nonameric peptide VMAPRTLIL derived from the UL40 protein of the human cytomegalovirus (HCMV), mimicking the signal peptide sequence present in most HLA-C allotypes [26]. Alterations of this peptide sequence have a strong impact on the recognition by CD94/NKG2A or NKG2C receptor and consequently have a high impact on NK cell cytotoxicity [27]. An example of peptide mediated cytotoxicity could be observed with the QMRPVSRVL peptide derived from HSP60, which showed total loss of recognition by the CD94/NKG2A inhibitory receptor [17], leading to cytotoxic NK cell responses due to the triggering by stimulatory receptors KIR2DS1 or NKG2D [28, 29]. These subtle alterations in the sequences of peptides bound to HLA-E impact extensively on the fine tuning of NK cell receptor binding and responses.

**Figure 1:**
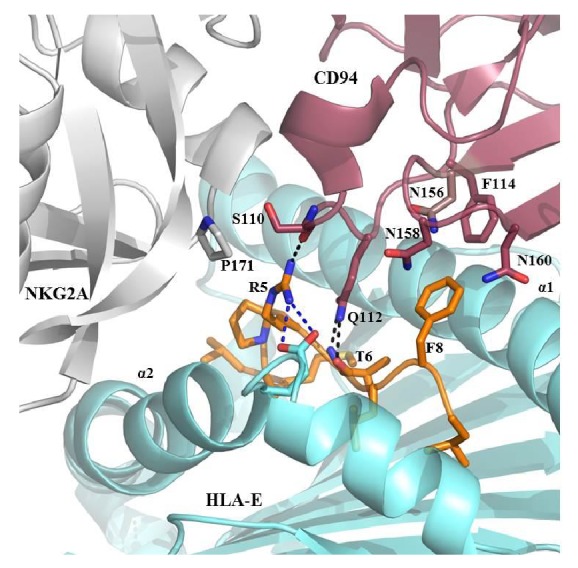
C-terminal peptide residues mediate contact between the HLA-E^VMAPRTLFL^ complex and the CD94/NKG2A receptor. This structure represents the interactions between the CD94/NKG2A receptor and the HLA-E^VMAPRTLFL^ complex (PDB: 3CDG) [4]. The CD94 subunit (raspberry) dominates the recognition of the peptide (orange sticks) with several contacts including hydrogen bonds of Ser110 to the peptide's p5-Arg and Gln112 to p6-Thr. The peptide's p8-Phe is surrounded by a polar pocket created by Asn156, Asn158, and Asn160 and van der Waals contacts with Phe114. p5-Arg also builds a salt bridge to Glu152 of the HLA-E heavy chain (pale teal) that may prevent more charged interactions with CD94/NKG2A. Residue Pro171 of NKG2A (pale grey) interacts with p5-Arg by van der Waals interactions. Hydrogen bonds are represented as black dashed lines, and salt bridges are given in blue.

The structural analysis of HLA-E^VMAPRTLFL^ in complex with the CD94/NKG2A receptor illustrates the impact of the peptide sequence, where an AA difference might result in the loss of the CD94/NKG2A receptor recognition [4] (Figure 1). The main contribution to direct interactions with the VMAPRTLFL peptide residues is mediated by the CD94 subunit through a hydrogen bond between CD94-Ser110 to the guanidinium group of the peptide's p5-Arg and a hydrogen bond of CD94-Gln112 to the main chain of the peptide's p6-Thr. The CD94-Gln112 additionally contacts p5-Arg and p8-Phe by van der Waals interactions. The p8-Phe is surrounded and contacted by the three polar CD94 residues Asn156, Asn158, and Asn160 and also interacts with Phe114. The NKG2A subunit of the CD94/NKG2A receptor complex exclusively contacts the peptide's p5-Arg with residue NKG2A-Pro171 through van der Waals interactions.

The impact of the peptide sequence on CD94/NKG2A receptor recognition was also shown with the peptide VMAPRALLL derived from HLA-Cw^*^07 : 02 signal peptide that resulted in highly reduced recognition compared to the p6-Thr variant by the CD94/NKG2A receptor and could therefore not protect from NK cell lysis [30]. Although the peptide's p6-Thr (VMAPRTLFL) is substituted by p6-Ala (VMAPRALLL), this substitution did not change the conformation of the HLA-E heavy chain or the orientation of exposed side chains at p5 or p8 [31]. The subtle fine tuning of immune responses is explained as a result of peptide sequence alterations of HLA-E bound ligands.

HLA-E also plays a role in adaptive immunity, since certain pHLA-E complexes are recognized by subsets of CD8^+^ T cells [32]. Evidence for a function of HLA-E in T cell immunity could be observed with several pathogen derived ligands; in this case, certain pHLA-E complexes could not be recognized anymore by CD56^+^/CD94^+^/NKG2A^+^ NK cells. Here a nonameric peptide SQAPLPCVL derived from the Epstein-Barr virus BZLF1 protein bound to HLA-E could be recognized by the *α* *β*-T cell receptor (TCR) from a CD94^+^/NKG2A^+^/CD8^+^ T cell clone [33]. Furthermore, it has been demonstrated that CD4^−^/CD56^−^/CD8^+^ T cells, isolated from individuals that were immunized with a* Salmonella enterica serovar Typhi* vaccine, are specifically activated by B-LCLs that were expressing recombinant HLA-E and loaded with* S. Typhi* derived peptides [34]. Since it is known that HLA-E binds to a peptide VMAPRTLIL derived from the HCMV UL40 protein and serves as a ligand for the CD94/NKG2A and CD94/NKG2C NK cell receptor, this HLA-E^VMAPRTLIL^ complex is also recognized by the TCR of CD45RA^+^/CD28^−^/CD27^−^CD56^+^ effector memory like T cells and leads to T cell mediated cytotoxicity [35].

The peptide-mediated fine tuning of immune responses within the innate immune system could be detected among subsets of the CD8^+^ T cell repertoire as well. The HCMV UL40 protein contains mutations among different HCMV strains, resulting in single AA exchanges within the peptide; consequently, a different subset of CD8^+^ T cells, specific for the complex HLA-E^VMAPRTLVL^ [36], where the peptide's p8-Ile is exchanged for a p8-Val, could be identified.

The peptide specific recognition by its cognate TCR could be analyzed in the crystal structure of HLA-E^VMAPRTLIL^ in complex with its cognate TCR, derived from a UL40 specific T cell clone. The affinity of this TCR to the HLA-E^VMAPRTLIL^ complex is relatively lower compared to TCR interactions with classical pHLA complexes [37] that resulted in a lower on-rate of the interaction between the TCR and the HLA-E molecule. However, the half-life of this TCR/HLA-E^VMAPRTLIL^ complex is comparable to classical TCR/HLA interactions [38]. Conformational changes of a pHLA-E molecule in complex with the cognate TCR showed comparable values to classical pHLA/TCR complexes. The mode of interaction between a TCR and a distinct pHLA-E complex could be explained by the importance of residue 8 within the VMAPRTLIL peptide. The contribution of p8-Ile to the binding of the TCR was shown to be crucial for a stable TCR/HLA-E^VMAPRTLIL^ complex. Alterations at position 8 of the peptide to either Val or Leu resulted in a significant reduction of affinity to the TCR [39].

The importance of regulatory functions of HLA-E molecules and its peptide ligands for the balancing regulation of innate and adaptive immune response is highlighted and should be considered in situations where normal HLA class I expression is reduced or abrogated. Taken together, the source of peptide determines the role of the given pHLA-E complex in innate or adaptive immunity.

## 3. HLA-E and Malignancies

Since HLA-E is an effective inhibitory molecule whose main role is to prevent NK cell activation, it is a useful protective molecule for malignant cells in order to prevent their fate from NK cell killing. The role of HLA-E in controlling NK cell activity in the context of viral interference could be shown recently by demonstrating how miR-376a(e) regulates HLA-E expression during HCMV infection [40]. The downregulation of HLA class I molecules is a widespread escape mechanism of tumor cells to prevent the recognition by CD8^+^ T cells [41, 42]. The overexpression of HLA-E on tumor cells has recently been reported in colorectal cancer and was pointed out as a biomarker for tumor cell differentiation [43]. This overexpression of HLA-E is proposed to be associated with the inhibition of tumor tissue infiltrating NK or CD94^+^/NKG2A^+^/CD8^+^ T cells, resulting as a poor prognosis marker. Studies on colorectal cancer cell lines showed accordingly an overexpression of HLA-E that was correlated with the malignancy stage and furthermore the release of soluble HLA-E molecules from these cell lines [44]. A protective role of HLA-E in tumor cells was also underlined in patients with ovarian and cervical cancer, where tumor infiltrating CD8^+^ cytotoxic T cells (CTLs) showed an upregulation of the CD94/NKG2A inhibitory receptor, whereas NK cells were only found at very low numbers in the tumor tissues. Regarding HLA-E expression, the benefit of tumor infiltrating CTLs was abrogated presumably due to the inhibition of CD94^+^/NKG2A^+^ CTLs by HLA-E [45, 46]. Furthermore, HLA-E expression in early breast cancer patients was also proposed as a prognostic marker for the outcome on tumor progression [47]. Despite the downregulation of HLA class I molecules during the tumor immune escape, the surface expression of HLA-E is not affected. A similar immune escape mechanism has been reported for human fibroblasts infected with HCMV, where HLA-E is presented on the cell surface associated with downregulation of HLA class I [48]. Viral proteins specifically target and downregulate several components of the peptide loading complex, such as transporter associated with antigen processing (TAP) or tapasin (TPN) [49, 50]. Recently we found a TPN-independent peptide loading mechanism for HLA-E variants [51]. This observation explains the role of HLA-E and its differential surface expression levels in malignancies. Considering the HLA-E genotype and the differences in surface expression of HLA-E^*^01 : 01 and HLA-E^*^01 : 03, the lower surface expression of HLA-E^*^01 : 01 could be a benefit for antitumor immune responses resulting in less inhibitory ligands for CD94^+^/NKG2A^+^ immune cells.

## 4. Influence of HLA-E on HSCT Outcome

Even though the highly important properties of HLA-E regarding its antigen presentation and recognition by effector cells are clear, its clinical relevance for transplantation outcome is still controversy. It has been demonstrated that HLA-E polymorphisms in 10/10 HLA matched unrelated donor and recipients had no significant impact on the outcome of a hematopoietic stem cell transplantation (HSCT) [52]. In contrast to this finding an association between HLA-E^*^01 : 03 homozygosity and a significant improvement for HSCT outcome was published [53]. Both studies analyzed cohorts of intermediate size (*N* = 116; 83) but also with differences in patient treatment specifications, subsidiary diseases, and followup. Another clinical study provided data, where homozygous HLA-E^*^01 : 03 grafts revealed a significant lower risk for acute and chronic graft versus host disease (aGvHD; cGvHD) associated with a postulated impaired efficiency of minor histocompatibility antigen presentation by HLA-E^*^01 : 03, but also a significant increased risk for transplant related mortality (TRM) [54].

A general conclusion for the role of HLA-E in HSCT is not feasible, yet, since only a limited amount of clinical data considering cohort size and differences in patient treatment as well is available, this needs to be completed by comprehensive further studies. However, the fact that HLA-E is also a target for a subset of CD8^+^ T cells contributes to its posttransplant role. The recognition by HLA-E restricted CD8^+^ T cells emerges to the spotlight that was shown in HCMV-seropositive transplant patients that restore a subset of HLA-E-reactive CD8^+^ T cells [55]. These CD8^+^ T cells developed cytolytic activities when cultured with different HLA-E haplotyped endothelial cell cultures independently of the HLA-E allele. This activation was observed not only for HCMV seropositive but also for seronegative endothelial cells. This indeed suggests the direct recognition of allogeneic HLA-E on graft endothelial cells in solid organ transplantation. However, the activation could be inhibited due to the presence of specific HLA-C haplotypes that are ligands for the classical HLA class I NK receptor KIR2DL2 [56]. This receptor was highly expressed on the HLA-E restricted CD8^+^ T cell subsets. This data suggests that HCMV-associated HLA-E-restricted T cells could contribute to allograft rejection in the case of HLA-C haplotype/NK receptor mismatch on graft tissue.

## 5. Conclusion

The ongoing studies on HLA-E antigen presentation and functional effects revealed that HLA-E is a bridge between innate and adaptive immunity. Early research proposed a very restricted peptide repertoire capable of binding to HLA-E. Since HLA-E is not exclusively restricted to canonical peptides of HLA class I origin, its immunogenic features are probably more important for transplantation outcome due to its broader spectrum of presented peptides. For a certain estimation of the influence of HLA-E on transplantation outcome, the focus of clinical studies on HLA-E restricted HCMV-associated CD8^+^ T cell subsets needs to be extended and compared in greater cohorts.

The recognition of HLA-E molecules by the TCR of certain CTLs could be a promising option in anticancer therapies, since HLA-E surface expression has been shown on several types of tumors. However, the generation of CTLs with a specific TCR raised against antigenic peptides that are not alloreactive against nontumor HLA-E expressing cells is a crucial part that needs to be carefully considered. For this, the identification and functional assessment of tumor specific peptides presented by HLA-E and the presumable differences in the peptide repertoire of the two functional HLA-E alleles are of high importance.

## Figures and Tables

**Table 1:** HLA-E peptide ligands.

Peptide ligands (origin)	HLA-E∗01:01	HLA-E∗01:03	TCR	CD94/NKG2A	Reference
Naturally presented ligands					
ALALVRMLI (ATP binding cassette transporter, MRP7)	ND	ND	ND	+	[18]
VMAPRTLFL (HLA-G)	+	+	−	+	[33]
VMAPRTLIL (CMV UL40; HLA-C)	+	+	+	+	[26, 35, 38]
VMAPRTLVL (CMV; HLA-A)	+	+	+	+	[36, 57]

Predicted ligands					
QMRPVSRVL (human HSP60)	+	+	ND	−	[17]
AISPRTLNA (HIV Gag protein)	ND	ND	ND	+	[19]
SQQPYLQLQ (gliadin-wheat protein)	ND	ND	ND	+	[20]
SQAPLPCVL (EBV-BZLF1 protein)	+	+	+	+	[33]

This table shows the diversity of naturally presented or predicted HLA-E peptide ligands and their interactions with immune receptors. Since CD94/NKG2C receptor interactions with HLA-E bound to noncanoncial peptide ligands are poorly reported in the literature, data is not included. +: positive for binding; −: no binding; ND: not determined.

## Abbreviations

HSCT: Hematopoietic stem cell transplantation
GvHD: Graft versus host disease
pHLA-E: Peptide-HLA-E complexes
AA: Amino acid
CTL: Cytotoxic T lymphocytes.
